# Prevalence and Distribution of Nonsyndromic Dental Anomalies in Children in Eastern Saudi Arabia: A Radiographic Study

**DOI:** 10.1155/2021/9914670

**Published:** 2021-10-06

**Authors:** Eman A. Bakhurji, Fatima Aldossary, Jood Aljarbo, Fatimah AlMuhammadi, Maysaa Alghamdi, Muhammad Ashraf Nazir

**Affiliations:** ^1^Department of Preventive Dental Sciences, College of Dentistry, Imam Abdulrahman Bin Faisal University, Dammam, Saudi Arabia; ^2^College of Dentistry, Imam Abdulrahman Bin Faisal University, Dammam, Saudi Arabia

## Abstract

**Objectives:**

To report the prevalence and distribution of nonsyndromic dental anomalies in children in eastern Saudi Arabia.

**Methods:**

This retrospective records review study involved radiographic examination of 6–18 years old pediatric patients who attended the Dental Hospital of the College of Dentistry, Imam Abdulrahman Bin Faisal University, Dammam, Saudi Arabia. Four calibrated examiners recorded nonsyndromic dental anomalies from patients' digital orthopantomograms (OPG). The anomalies recorded were related to tooth number, shape, and location. Descriptive statistics, chi-square test, and Fisher exact tests were used to report prevalence and differences by gender, nationality, and medical history at the 5% significance level.

**Results:**

Of 2226 reviewed patients' records, 1897 met the inclusion criteria and were included in the study. The study sample had equal distribution of males (52.6%) and females (47.4%) and comprised 81.2% Saudi children with a mean age of 8.8 ± 1.84 years. Most study subjects (97.8%) were in mixed dentition and 88.7% were healthy. The prevalence of dental anomalies was as follows: teeth rotations (24.5%), ectopically erupted teeth (6%), congenitally missing permanent teeth (5.4%), peg lateral (1.1%), supernumerary (0.5%), gemination (0.3%), and fusion (0.1%). No statistically significant differences were found in the distribution of dental anomalies by patients' gender, medical history, and nationality (*p* value ≥0.05).

**Conclusion:**

This study showed that teeth rotations were the most common dental anomalies followed by ectopic eruptions and congenitally missing teeth. The study findings may guide dental practitioners to better diagnose and manage children with dental anomalies in eastern Saudi Arabia.

## 1. Introduction

Dental anomalies are deviations from the natural tooth location, number, and shape that occur during or after tooth formation [1, 2]. Anomalies of tooth shape and morphology include, but not limited to, gemination, fusion, and peg lateral. Congenitally missing and supernumerary teeth represent anomalies of number, while ectopic eruption and rotation of teeth are considered anomalies of location [2]. The etiology of dental anomalies is multifactorial and complex; it could be congenital, developmental, or acquired [2, 3].

The literature shows that dental anomalies can lead to malocclusion problems, aesthetic deformities, speech problems, poor oral hygiene, caries, and periodontal diseases which adversely affect the quality of life [1, 4–6]. Moreover, dental anomalies in primary dentition increase the likelihood of their presence in permanent dentition [7]. Studies conducted in the different regions of the world reported varying prevalence estimates of dental anomalies in children possibly because of ethnic differences, sampling methods, and diagnostic criteria [4, 6–10]. Other international studies reported the distribution of dental anomalies among children and adults. [11, 12].

In the Gizan region of Saudi Arabia, Salim reported the distribution of congenitally missing teeth in 2.2% of Saudi children followed by supernumerary teeth (0.50%) and peg-shaped lateral incisors (0.37%) [13]. Osuji and Hardie recorded congenitally missing teeth in 3.6% of children in Tabuk, Saudi Arabia [14]. In 2012, Afify and Zawawi also reported that congenitally missing teeth (25.7%) were the most common dental anomalies among Saudi patients (12–32 years) in the western region of Saudi Arabia [15]. Yassin in 2016 conducted a study in the southwest region of the country and concluded that the most prevalent dental anomaly was congenitally missing teeth (9.7%) among Saudi children [1]. Researchers also reported dental anomalies in predominately adult patients in different parts of Saudi Arabia [16–18]. Al-Jabaa and Aldrees observed a higher prevalence of dental anomalies among patients with malocclusion than patients without malocclusion in Riyadh, Saudi Arabia [19].

Dental anomalies may require complex and multidisciplinary management approaches in pediatric patients; therefore, understanding the prevalence of dental anomalies is crucial for early diagnosis and proper treatment planning. However, there is a lack of evidence on the prevalence of dental anomalies in children in the Eastern Province of Saudi Arabia. This study aimed to determine the prevalence and distribution of dental anomalies in children in the Eastern Province of Saudi Arabia using radiographic records. The study also investigated the association of dental anomalies with gender, nationality, and medical history.

## 2. Methods

This retrospective records review study included patient records of children visiting the Dental Hospital at the College of Dentistry, Imam Abdulrahman Bin Faisal University (IAU), Dammam, Saudi Arabia. The Scientific Research Unit at the College of Dentistry, IAU, approved the study (Ethical Approval # 202101)

A total of 2226 pediatric patients' records over the past 5 years (from the year 2015 to 2020) were identified and reviewed. For these records to be included in the study, the following inclusion criteria had to be met: (1) patient should be in mixed or permanent dentition, (2) with an age range of 6–18 years, and (3) should have had digital orthopantomograms (OPGs). Patients with previous trauma to the head, trauma that caused loss of teeth, missing teeth due to extraction, and patients with syndromes or conditions that are known for dental anomalies were excluded from the study. The initial digital OPGs were examined, and the patients' charts were reviewed to rule out the history of tooth extraction. All patients' identifiable information (name and ID) were not recorded, and confidentiality was maintained.

Training and calibration of four reviewers were done prior to the conduct of the study over 3 consecutive calibration sessions. The training involved radiographic cases (OPG) of different dental anomalies. Reviewers answers were compared to a gold standard pediatric dentist by kappa statistics for inter and intraexaminer reliability. Agreement was substantial (kappa ≥ 0.8). The radiographic examination of the patients' digital OPGs was carried out in a standardized method using the MiPACS Dental Enterprise Viewer software. Magnification option in the software was used when needed. The anomalies were recorded if they were identified from the radiographic records. These anomalies were divided into three categories: anomalies in the tooth number (supernumerary and congenitally missing teeth), anomalies in the shape (peg lateral, gemination, and fusion), and anomalies in the position (rotation and ectopic eruption).

Demographical data (gender, age, nationality, medical history, and type of dentition at time of initial taking of the OPG) were recorded for each patient based on the information available on their files. The nationality of the subjects was used as a proxy of patients' ethnic background, since the ethnicity of patients is not usually recorded in the patients' files. Significant medical history was considered if the patient has any medical condition recorded on file including medications. Radiopacities in tooth form and nontooth form were considered supernumerary including mesiodenses. Bilateral congenitally missing teeth were considered if the crown formation has occurred for the patient's age. Unilateral missing tooth was reported if the crown of the contralateral tooth has completely formed. Missing third molars were not considered as congenitally missing teeth. The ectopic eruption was recorded if the erupting tooth was in contact with an erupted tooth that demonstrated resorption. Rotation was considered if the tooth was repositioned by turning it on its long axis. Fusion was reported when separate pulp chambers join at the dentin level and the patient has fewer teeth. Gemination was reported when the tooth appeared to have one pulp canal, but two pulp chambers and teeth number are normal [20].

Data were analyzed using the Statistical Package for the Social Sciences (SPSS) version 25.0.0.2 (IBM Corp., Armonk, NY, USA) at 5% significance level. Frequencies and percentages were calculated to record the prevalence, location, and the number of each anomaly per patient. Chi-square and Fisher exact tests were used to report on the difference in the prevalence of each anomaly by the patient's gender, medical history, and nationality.

## 3. Results

Of 2225 reviewed patients' records, only 1897 records (85.3%) met the inclusion criteria and were included in the analysis of this study. The study sample had almost equal distribution of males (52.6%) and females (47.4%). However, most subjects were Saudis (81.2%) and had no significant medical history (88.7%). The age range of the children was between 6 and 18 years, and the mean age was 8.8 ± 1.84 years at the time of taking the OPGs (Table 1).

Table 2 provides the prevalence and distribution of all anomalies in the study subjects. The prevalence of supernumerary teeth in children was 0.5%, and most of the children involved in the study had only one supernumerary tooth (88.9%) and had it located in the anterior region of the mouth (77.8%). On the contrary, the prevalence of congenitally missing permanent tooth was 5.4%. Forty-one percent of the subjects had one congenitally missing tooth, while 48% had two or more congenitally missing teeth. Two-thirds of these teeth were in the posterior area of the mouth (62.1%) and one-third of them were in the anterior region. With regards to anomalies related to the shape of teeth, peg lateral had the highest prevalence (1.1%), while the prevalence of gemination was 0.3% and fusion was 0.1%. Tooth rotation (anomaly of tooth position) had the highest prevalence (24.5%) among all anomalies, and the majority of teeth were in the anterior area (87%). On the other hand, the prevalence of ectopically erupted permanent teeth was 6%.

Table 3 provides differences in the distribution of anomalies by patients' gender, medical history, and nationality. No statistically significant differences were noted in any of the anomalies based on patients' gender, nationality, or medical history (*p* value ≥0.05).

## 4. Discussion

The present study aimed to evaluate dental anomalies among children in the eastern region of Saudi Arabia. The study found that about one-quarter of children had teeth rotation which was the most common dental anomaly in our sample. This finding is in accordance with the results of a study by Vani et al. where teeth rotation was observed in 20.2% of subjects attending the dental clinics of the College of Dentistry, Jazan University, Jazan, Saudi Arabia [17]. In contrast, Yassin observed teeth rotation in 1.6% of patients who visited the dental clinics at King Khalid University College of Dentistry, Abha, Saudi Arabia [1]. Similar to our study, Gupta et al. reported that teeth rotation was the most prevalent (10.24%) dental anomaly in Indian population [21]. Similarly, Kathariya et al. showed teeth rotation in 13.17% of Indian subjects [22]. It is known that teeth rotations can result from disturbances during preeruptive and posteruptive phases because of trauma, cysts, tumors, tooth extractions, ectopically erupted teeth, hypodontia, and supernumerary teeth [1, 17, 23].

The etiology of ectopic eruption is not well known, but it includes genetic and local factors. Local factors include small arches, early eruption of maxillary first permanent molar, insufficient anteroposterior growth of the jaws, bone growth at tuberosity area, deviation in the path of eruption, and abnormal crown morphology of primary second molar [24, 25]. The ectopic eruption was the second most common (6%) dental anomalies in the present study. This finding is supported by the results of studies conducted locally and internationally [17, 21]. A previous study from Saudi Arabia reported ectopic eruptions in 7.6% of patients, and this was the second most frequent dental anomaly [17]. However, Afify and Zawawi observed ectopic eruption only in 0.3% of subjects who attended the Faculty of Dentistry, King Abdulaziz University, Jeddah, Saudi Arabia [15]. The results of a study from India showed ectopic eruption in 7.93% of subjects, and this was the second most common dental anomaly [21].

The literature cites that congenitally missing teeth are the most prevalent dental anomaly which can lead to aesthetic and functional complications requiring expensive multidisciplinary treatment [26]. The prevalence and type of congenitally missing teeth vary among different ethnic groups [1, 15]. Previous studies reported prevalence estimates of congenitally missing teeth ranging from 2.2 to 9.7 among children in Saudi Arabia [1, 13, 14]. According to the international literature, congenitally missing teeth were among the most frequent dental anomalies in children in Slovenia (7.2%), Rome (7.1%), and Turkey (3.67%) [8, 26, 27]. On the other hand, congenitally missing permanent teeth (5.4%) were the third most common dental anomalies in the present study.

The three least common dental anomalies included fusion (0.1%), gemination (0.3%), and supernumerary teeth (0.5%) in our sample of children. The prevalence of fusion varies depending upon geographic, racial, or genetic factors. Only 0.8% of children in Saudi Arabia were shown to have fused teeth [1]. Similar prevalence estimates of fusion (0.5%) were reported in a study of children in India [28]. Regarding the distribution of geminated teeth, a previous study showed gemination in 0.3% of children in Sweden which is consistent with our study results [29]. Similarly, only 0.08% of children in Saudi Arabia were shown to have geminated teeth [13].

Supernumerary teeth are those which develop in addition to the normal dentition, and they can cause cystic lesions, diastema, dental impaction, delayed eruption, and crowding [8, 19]. The present study showed a low prevalence of supernumerary teeth. Other studies from different regions of Saudi Arabia also demonstrated low distribution (0.50–3.5%) of supernumerary teeth in children [1, 13]. Low prevalence of supernumerary teeth was also noted among Swedish children (1.9%) by Bäckman and Wahlin [29], Italian children (0.9%) by Laganà et al. [8], Turkish children (0.96%) by Karadas et al. [27], and Greek children (1%) by Pallikaraki et al. [30]. Overall, wide variations in the distribution of dental anomalies reported in the literature may be attributed to sample size, age of study subjects, and diagnostic criteria in addition to genetic and racial factors.

A previous study reported no significant differences in dental anomalies between male and female children in Saudi Arabia [1]. A similar finding of no significant difference in dental anomalies between the gender was shown in Turkish children [27]. Likewise, the distribution of dental anomalies between male and female children did not differ significantly in the present study. In addition, dental anomalies in the present study showed significant differences based on the nationality and medical history.

This study is the first to report the distribution of dental anomalies among children in the Eastern Province of Saudi Arabia. Reporting on different dental anomalies in the area is crucial for the provision of appropriate diagnosis and management of these anomalies. For example, in this study, the prevalence of rotated teeth was the highest among all anomalies. This information would be helpful to clinicians while examining pediatric patients to look for this specific common problem and be better prepared to manage it. Data accuracy was given utmost importance during the study by calibrating four examiners with excellent inter and intraexaminer reliability.

There are certain limitations that should be considered while interpreting the study results. The study did not report information on the patient's ethnic background because this information is not usually recorded in the patient's medical record. However, the nationality of the patients was considered as a proxy of their different ethnic backgrounds. The study used a large data of children visiting a public dental hospital in the eastern region; therefore, results from this study are representative of patients with the similar age group residing in the area. However, generalizing the study results to children attending other dental hospitals in different regions of the country should be avoided. Future research should use a nationally representative sample of children for the investigation of dental anomalies.

## 5. Conclusion

The present study showed that teeth rotations were the most common dental anomaly followed by ectopic teeth eruptions and congenitally missing permanent teeth. On the other hand, fusion, gemination, and supernumerary teeth were the least common dental anomalies. Dental anomalies did not differ significantly by patients' gender, nationality, and medical history. The study findings may guide dental practitioners to better diagnose and manage children with dental anomalies in eastern Saudi Arabia.

## Figures and Tables

**Table 1:** Demographic distribution of study subjects (*N* = 1897).

Variables	*N* (%)
Gender	
Male	997 (52.6)
Female	900 (47.4)

Nationality	
Saudi	1540 (81.2)
Non-Saudi	357 (18.8)

Medical history	
None	1683 (88.7)
Medically compromised	214 (11.3)

Dentition at the first visit	
Mixed	1856 (97.8)
Permanent	41 (2.2)

Age at time of radiographs	Mean ± SD
	8.8 ± 1.84
	Range: 6–18 years old

**Table 2:** Prevalence and distribution of anomalies according to teeth number, shape, and location among children (*N* = 1897).

*(1) Prevalence of anomalies according to number of teeth, n (%)*	
Supernumerary teeth: prevalence	9 (0.5)
Location	
Anterior	7 (77.8)
Posterior	1 (11.1)
Both	1 (11.1)

Number of supernumeraries per patient	
One	8 (88.9)
≥2	1 (11.1)

Congenitally missing permanent teeth: prevalence	103 (5.4)
Location	
Anterior	34 (33.0)
Posterior	64 (62.1)
Both	5 (4.9)

Number of congenitally missing permanent teeth per patient	
One	42 (40.8)
≥2	61 (59.2)

*(2) Prevalence of anomalies according to shape of teeth, n (%)*	
Gemination: prevalence	5 (0.3)
Number of geminated teeth per patient	
One	5 (100)
≥2	0

Fusion: prevalence	1 (0.1)
Number of fused teeth per patient	
One	1 (100)
≥2	0

Peg lateral: prevalence	20 (1.1)
Number of peg laterals per patient	
One	16 (80)
≥2	4 (40)

*(3) Prevalence of anomalies according to position of teeth, n (%)*	
Rotation: prevalence	464 (24.5)
Location	
Anterior	405 (87.3)
Posterior	38 (8.2)
Both	(4.5)

Number of rotated teeth per patient	
One	262 (56.5)
≥2	202 (43.5)

Ectopic eruption: prevalence	113 (6)
Number of ectopic-erupted teeth per patient	
One	86 (76.1)
≥2	27 (23.9)

**Table 3:** Distribution of dental anomalies by gender, medical history, and nationality among children (*N* = 1897).

*Gender, n (%)*			
Anomalies	Male	Female	*P* value
Supernumerary teeth	5 (55.6)	4 (44.4)	≥0.05 ^*∗*^
Congenitally missing permanent teeth	48 (46.6)	55 (53.4)	≥0.05
Gemination	2 (40)	3 (60)	≥0.05 ^*∗*^
Fusion	1 (100)	0 (0)	≥0.05 ^*∗*^
Peg lateral	10 (50)	10 (50)	≥0.05
Ectopic eruption	61 (54)	52 (56)	≥0.05
Rotation	249 (53.7)	215 (46.3)	≥0.05

*Medical history, n (%)*			
Anomalies	None	Medically compromised	*P* value
Supernumerary teeth	7 (77.8)	2 (22.2)	≥0.05 ^*∗*^
Congenitally missing permanent teeth	92 (89.3)	11 (10.7)	≥0.05
Gemination	4 (80)	1 (20)	≥0.05 ^*∗*^
Fusion	1 (100)	0 (0)	≥0.05 ^*∗*^
Peg lateral	18 (90)	2 (10)	≥0.05 ^*∗*^
Ectopic eruption	96 (85)	17 (15)	≥0.05
Rotation	402 (86.6)	62 (13.4)	≥0.05
Nationality, *n* (%)			
Anomalies	Saudi	Non-Saudi	*P* value
Supernumerary teeth	6 (66.7)	3 (33.3)	≥0.05 ^*∗*^
Congenitally missing permanent teeth	81 (82.9)	22 (17.1)	≥0.05
Gemination	4 (80)	1 (20)	≥0.05 ^*∗*^
Fusion	1 (100)	0 (0)	≥0.05 ^*∗*^
Peg lateral	16 (80)	4 (20)	≥0.05
Ectopic eruption	92 (81.4)	21 (18.6)	≥0.05
Rotation	379 (81.7)	85 (18.3)	≥0.05

^*∗*^Fisher exact test.

## Data Availability

The data used to support the findings of this study are available from the corresponding author upon request.
